# Binding, Thermodynamics, and Selectivity of a Non-peptide Antagonist to the Melanocortin-4 Receptor

**DOI:** 10.3389/fphar.2018.00560

**Published:** 2018-06-01

**Authors:** Noureldin Saleh, Gunnar Kleinau, Nicolas Heyder, Timothy Clark, Peter W. Hildebrand, Patrick Scheerer

**Affiliations:** ^1^Charité – Universitätsmedizin Berlin, Corporate Member of Freie Universität Berlin, Humboldt-Universität zu Berlin and Berlin Institute of Health, Institute of Medical Physics and Biophysics, Berlin, Germany; ^2^Computational Modelling and Dynamics of Molecular Complexes, Berlin, Germany; ^3^Group Protein X-ray Crystallography and Signal Transduction, Berlin, Germany; ^4^Computer-Chemie-Centrum, Department of Chemistry and Pharmacy, Friedrich-Alexander University Erlangen-Nürnberg, Erlangen, Germany; ^5^Institute of Medical Physics and Biophysics, Leipzig University, Leipzig, Germany

**Keywords:** MC4R, melanocortin-4 receptor, melanocortin-receptors, ligand binding, ligand selectivity, molecular dynamics

## Abstract

The melanocortin-4 receptor (MC4R) is a potential drug target for treatment of obesity, anxiety, depression, and sexual dysfunction. Crystal structures for MC4R are not yet available, which has hindered successful structure-based drug design. Using microsecond-scale molecular-dynamics simulations, we have investigated selective binding of the non-peptide antagonist MCL0129 to a homology model of human MC4R (hMC4R). This approach revealed that, at the end of a multi-step binding process, MCL0129 spontaneously adopts a binding mode in which it blocks the agonistic-binding site. This binding mode was confirmed in subsequent metadynamics simulations, which gave an affinity for human hMC4R that matches the experimentally determined value. Extending our simulations of MCL0129 binding to hMC1R and hMC3R, we find that receptor subtype selectivity for hMC4R depends on few amino acids located in various structural elements of the receptor. These insights may support rational drug design targeting the melanocortin systems.

## Introduction

The agonistic proopiomelanocortin-derived neuropeptides α-, β-, and γ-melanocyte-stimulating hormone (α-, β-, γ-MSH), the adrenocorticotropic hormone (ACTH) and the inverse agonistic agouti-related peptide (AgRP) are ligands for five closely related melanocortin receptors (MC1-5R), a subgroup of class A G-protein coupled receptors (GPCRs) (Gantz et al., 1993; Cone, 2005, 2006; Dores et al., 2014). The melanocortin system is involved in a plethora of physiological functions, including pigmentation, steroidogenesis, energy homeostasis, and sexual function (Cone, 2006; Rodrigues et al., 2015).

MC1R is expressed by the dermal melanocyte and regulates the synthesis of eumelanin (black–brown pigment) versus phaeomelanin (yellow–red pigment) (Herraiz et al., 2017). The ligand ACTH acts on MC2R (Liang et al., 2013) and is involved in controlling glucocorticoid levels (Fridmanis et al., 2017). The exact physiological role of the MC3R is not clear. Potential roles include control of energy homeostasis in addition to MC4R or immune response and circadian rhythms (Girardet et al., 2014; Mountjoy, 2015). Mice with targeted deletion of MC5R exhibit a defect in water and thermoregulation (Chen et al., 1997), and recent studies have suggested MC5R/α-MSH involvement in modulation of muscle glucose uptake and thermogenesis (Enriori et al., 2016).

The MC4R is of specific interest in the central melanocortin pathways, because it is known to regulate food intake, glucose homeostasis, and energy expenditure (Yang and Tao, 2017). The huge number of pathogenic heterozygous inactivating MC4R mutations are the most frequent genetic cause of obesity in humans (Farooqi et al., 2003). Consequently, MC4R agonists have been developed as potential therapeutics for obesity (Kievit et al., 2013; Chen et al., 2015; Kuhnen et al., 2016) and have been proven effective in treatment of male and female sexual dysfunctions, whereas MC4R antagonists are potential anxiolytics that may function as antidepressants or for treating cancer-induced anorexia (Chaki et al., 2003; Ercil et al., 2005; Pontillo et al., 2005; Chaki and Okubo, 2007; Lansdell et al., 2010).

Although several clinical trials with MC4R ligands have been initiated (reviewed in Ericson et al., 2017b), none of these ligands has yet been therapeutically approved, mainly because of adverse side effects related to low subtype or signaling selectivity (Yang and Harmon, 2017; Yang and Tao, 2017). For instance, the non-selective agonist bremelanotide has been shown to induce elevated blood pressure in clinical trial for male and female sexual dysfunction, while the MC4R selective peptide agonist setmelanotide (10-fold over MC3R) have shown no adverse effects on either blood pressure or heart rate in an anti-obesity study (Ju et al., 2018). However, setmelanotide treatment has impact on skin or hair coloring (Kuhnen et al., 2016). Additionally, MC4R agonist melanotan-II, a super-potent cyclic melanotropic peptide, increased blood pressure (Dorr et al., 1996). Animal models have revealed that MC4R and not MC3R, to be the receptor subtype responsible for stress-induced anorexia (Chaki and Okubo, 2007) and more recently an animal study provided a preclinical proof of concept of the HS014 selective antagonist in treating post-traumatic stress disorder (Sabban and Serova, 2018).

Generally, MC4R-targeting molecules can be linear or cyclic peptides, or small molecules (Goncalves et al., 2018). A comprehensive understanding of molecules that act at MC4R may help to design safe and effective anti-obesity drugs (Ju et al., 2018). Recent medicinal chemistry efforts have focused on using cyclized peptides, cyclotides, and constrained tetrapeptides to improve bioavailability, selectivity, and blood-brain-barrier uptake (reviewed in Zhou and Cai, 2017), whereas more recent small-molecule ligands development has focused on screening GPCR-privileged pharmacophores (reviewed in Ericson et al., 2017a).

Such drug-development problems have been solved for other GPCRs with the aid of robust structural information and structure-based drug design (SBDD) (Sabban and Serova, 2018). Whilst the last decade has witnessed a revolution in the field of GPCR structural biology (Granier and Kobilka, 2012), structural information regarding the melanocortin receptors and ligands has been limited to NMR data of AgRP or standalone transmembrane helix 2 (TM2) of the receptor (Sabban and Serova, 2018). Biomolecular [molecular dynamics (MDs)] simulations have been successful in providing missing structural information for GPCRs (Dror et al., 2011, 2012, 2013; Grossfield, 2011; Kruse et al., 2012; Vanni and Rothlisberger, 2012; Rose et al., 2014; Clark, 2017). Moreover, homology models based on high-quality templates in combination with microsecond-scale unbiased MD refinement (Clark, 2017) have provided robust experimentally testable structural and thermodynamic information on GPCRs (Milanos et al., 2016; Saleh et al., 2016, 2017a,b,c).

Using an established metadynamics protocol, we have been able to predict GPCR-ligand binding -affinities, -modes and free-energies accurately (Saleh et al., 2017b). Metadynamics simulations use a history-dependent bias to encourage the system to explore other areas of the free-energy landscape that are not accessible using plain MD simulation, even using special purpose supercomputers (Laio and Gervasio, 2008). Here, we have used microsecond-scale molecular-dynamics and metadynamics enhanced sampling to investigate binding mechanisms and thermodynamics of the selective non-peptide antagonist MCL0129 to human hMC4R.

These insights were used to unravel the molecular determinants of MCR subtype selectivity and the antagonistic properties of MCL0129.

## Materials and Methods

### Homology Modeling of the Initial hMC4R Conformation in an Inactive State

Human MC4R (hMC4R) is characterized by several specific properties with respect to the amino acid constitution and related structural features. In particular:

(i) an extremely short second extracellular loop 2 (ECL2, constituted by approximately four amino acids),
(ii) a missing cysteine disulfide bridge between the ECL2 and TM3 which is highly conserved among GPCRs,
(iii) a regular α-helical conformation of TM5 because of a methionine instead of a proline (Pro^5.50^) that is highly conserved in class A GPCRs (Supplementary Figure S1) and induces a helical kink and bulge,
(iv) the highly conserved class A GPCR motif ^7.49^*NP*xx*Y* in TM7 is a ^7.49^*DP*xx*Y* motif,
(v) a disulfide bridge in the third extracellular loop (ECL3) (Tarnow et al., 2003).

Because these features are of functional importance, we searched for a structural template that best represents the criteria described. Using the *GPCR-SSFE 2.0* modeling server (Worth et al., 2017), we found the inactive-state structure of the lyso-phospholipid sphingosine 1-phosphate receptor [S1PR1, PDB entry 3V2Y (Hanson et al., 2012)] with a sequence similarity in the transmembrane region of 56% and a sequence identity to hMC4R of 30% (Blossum62 matrix, Supplementary Figure S1). Such high sequence similarity between the suggested template and MC4R is important for the accuracy of the structural model (Costanzi et al., 2016). The template structure is characterized by a leucine at position 5.50 in TM5 (Ballesteros and Weinstein, 1995) and in consequence shows a regular α-helical conformation, as also expected for hMC4R. Moreover, the disulfide bridge between ECL2 and TM3 is missing but a disulfide bridge is located in the position proposed for MC4R (Sabban and Serova, 2018) (Supplementary Figure S1). The N-terminus of S1PR1 is 40 amino acids long, in comparison to 39 amino acids in hMC4R. The template structure starts with residue Ser17, the final MC4R model contains amino acids Asn17-Pro321.

**FIGURE 1:**
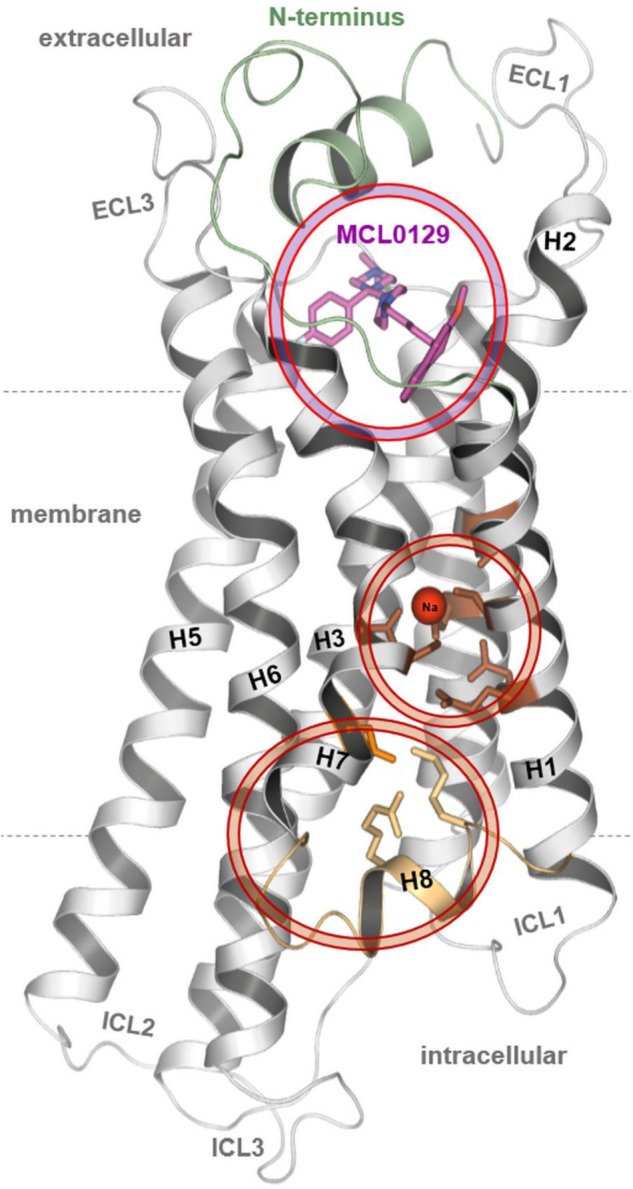
The final conformation of the hMC4R model with docked MCL0129, sodium binding and an unusual organization for the distal end of helices 7–8.

Structural modifications to generate a human MC4R homology model were performed with the software Sybyl X2.0 (Certara, Princeton, NJ, United States). For template preparation, the T4-lysozyme and the co-crystallized ligand were removed, loop lengths were adjusted by deletion of template amino acids (e.g., for ECL2) and gaps of missing residues in the loops of the template structure were closed manually (e.g., in ICL2 and ICL3). Moreover, missing residues between the N-terminal helical part and TM1 (between Ala39-Leu47) were added manually. Amino acids of the receptor-template were substituted with residues of human MC4R according to a sequence alignment between S1PR1 and hMC4R (Supplementary Figure S1), followed by conjugate gradient minimization of side chains until convergence to a gradient of 0.1 kcal/mol.Å with constraint backbone atoms of the transmembrane helices. This preliminary model was refined by MD simulations (300 K, 2 ns) of side chains and loops, followed by energy minimization of the entire model until convergence to a gradient of kcal/mol.Å. The quality and stability of the model (**Figure 1**) were validated by checking the geometry with the program PROCHECK (Laskowski et al., 1993).

### General Setup of the MD Simulations

Melanocortin receptor models were inserted into a 1-palmitoyl-2-oleoylphosphatidylcholine (POPC) membrane bilayer according to the orientation in the OPM database (Lomize et al., 2006). Parameters for the simulations were generated using the CHARMM-GUI web interface (Jo et al., 2008; Wu et al., 2014; Lee et al., 2016) using the CHARMM36 force field (Huang and MacKerell, 2013) with CgenFF (Vanommeslaeghe et al., 2010) for ligands with partial charges generated from AM1-BCC calculations (Jakalian et al., 2002). Ligand molecules were added 7 Å above the receptor’s extracellular surface. The appropriate number of sodium and chloride ions was added to the systems to simulate a physiological salt concentration of 150 mM. Particle-mesh Ewald (PME) (Darden et al., 1993) was used to treat electrostatic interactions, using a cut-off distance of 10 Å. The resulting system was geometry-optimized and then equilibrated for 10 ns followed by a production run. All simulations used the TIP3P water model. All simulations were performed using GROMACS 5.0.4 (Pronk et al., 2013) and the Plumed plug-in 2.1 (Tribello et al., 2014) for the metadynamics simulations. The simulation systems compromised a box of 75 Å × 75 Å × 110 Å.

Metadynamics simulations were performed following a recently published protocol (Saleh et al., 2017b). We used a combination of the well-tempered variant (WT) (Laio and Parrinello, 2002; Barducci et al., 2008) of metadynamics and funnel metadynamics (FM) (Limongelli et al., 2013). A metadynamics history-dependent bias was applied along the component of the *z*-distance between the relatively immobile C_α_ of Trp^6.48^ deep in the ligand-binding region and the center of mass of positively charged nitrogen atoms of MCL0129. This distance was used as the single collective variable. The funnel restraint was then applied to the relative position on the *xy*-plane (restrained to 8 Å radius when fully unbound and allowing 13 Å radius if around the vestibule or deeper) to ensure better sampling for the relevant region of the free energy, because the ligand can otherwise move extensively in the extracellular solvent without affecting the free energy. Gaussian hills with an initial height of 0.48 kcal mol^-1^ were applied every 1 ps. The hill width was chosen to be 1 Å. The Gaussian functions were rescaled in the WT scheme using a bias factor of 20.

Representative structures were extracted along the simulation of initial docking for each 2 Å window, and used as starting coordinates for the multiple walker technique (Raiteri et al., 2006). This ensured faster convergence of the free-energy surface and enhanced the parallelization. Free energies were calculated using the PLUMED plug-in (Tribello et al., 2014), as described in our protocol (Saleh et al., 2017b).

### Homology Modeling of the Inactive State hMC1R and hMC3R Structures

It is reported that the ligand MCL0129 is selective among the hMCR subtypes (Sabban and Serova, 2018) and we identified residues in the suggested hMC4R ligand binding site that are similar, identical, or specific compared to hMC1R, hMC2R, hMC3R or hMC5R (Supplementary Figure S1). The diverse amino acids may help explain ligand selectivity between the MCR subtypes; to test this hypothesis we also performed binding dynamics with MCL0129 at hMC1R and hMC3R. The hMC1R and hMC1R models were built based on the final hMC4R/MCL129 docking complex that resulted from the 5 μs unbiased simulation. For this purpose, the ligand was deleted from the hMC4R/MCL0129 complex. Missing residues [such as in hMC3R position Asp110 (ECL1) or Pro226-Ala227 (ICL3)] were inserted manually. Amino acids of the hMC4R model were substituted with corresponding residues of human hMC1R and hMC3R, respectively, according to the sequence alignment (Supplementary Figure S1), followed by conjugate-gradient minimization of side chains until converging at a termination gradient of 0.1 kcal/mol.Å with constraint backbone atoms of the transmembrane helices. These initial hMC1R and hMC3R models were refined by MD simulations of side chains and loops (300 K, 1 ns), followed by energy minimization of the entire model until convergence to a gradient of 0.05 kcal/mol.Å.

## Results

In this study, spontaneous binding of the selective MC4R non-peptide antagonist MCL0129 (1-[(1S)-1-(4-fluorophenyl)-2-[4-[4-(2-methoxynaphthalen-1-yl)butyl] piperazin-1-yl]ethyl]-4-propan-2-ylpiperazine) was observed during 5 μs unbiased MD simulation (Chaki et al., 2003) (see section “Materials and Methods”). These simulations were analyzed to

(i) elucidate the pathway and mechanism of MCL0129 binding to hMC4R,
(ii) detect binding intermediates and calculate binding affinities of MCL0129 using metadynamics simulations and to
(iii) explore the basic structural features of hMC4R in a stabilized antagonist-bound inactive state.

### Structural Features of the hMC4R Homology Model

The initial hMC4R model is based on the crystal structure of a lipid GPCR, the sphingosine 1-phosphate receptor 1 (S1PR1, PDB entry 3V2Y) (Hanson et al., 2012), which shares high sequence similarity (55%) and identity (30%) with hMC4R in the transmembrane region (Supplementary Figure S1). Strikingly, both receptors lack two prominent structural features: *(a)* Pro^5.50^ in TM5 and *(b)* a disulfide bridge between TM3 and ECL2. The absence of Pro^5.50^ [superscripts are according to the Ballesteros and Weinstein numbering scheme (Ballesteros and Weinstein, 1995)] (Supplementary Figure S1), which causes a kink and a bulge in TM5 of most class A GPCRs (Worth et al., 2017) [as in known structures of adrenergic-receptors (Sabban and Serova, 2018) and discussed in detail by Sansuk et al. (2011)], leads to a straight helix conformation in S1PR1 (Troupiotis-Tsailaki et al., 2017).

**FIGURE 2:**
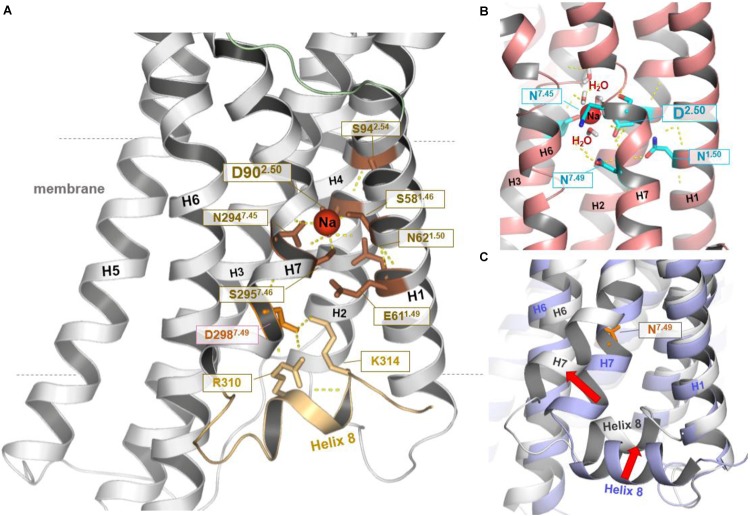
Structural–functional specificities observed in the hMC4R model. In the resulting hMC4R homology model **(A)** formation of a stable sodium binding pocket constituted by hydrophilic amino acid residues Ser^1.46^ (Ser58, TM1), Asp^2.50^ (Asp90, TM2), Ser^7.46^ (Ser295, TM7), and Asn^7.45^ (Asn294, TM7) was observed (dotted yellow lines indicate potential hydrogen bonds), surrounded by further hydrophilic residues in TM1 (Asn62, Glu61) and in TM2 (Ser94). Sodium binding under participation of the highly conserved amino acid Asp^2.50^ was already reported for the determined structures of protease-activated receptor (PAR) 1 [PDB entry 3VW7 (Zhang et al., 2012)] and PAR2 [PDB entry 5NDD (Cheng et al., 2017)], or as shown in **(B)** for the high-resolution structure of the A2AR [PDB entry 4EIY (Liu et al., 2012)]. Potential water molecules additionally surrounding the Na^+^ ion in a cluster like arrangement. We further note that during our MD simulations **(C)** the C-terminus of TM7 moved (∼5.6 Å) toward the membrane (red arrow, MC4R white backbone) in comparison to the template structure of TM7 (blue backbone-ribbon of S1PR1). This process was accompanied by an inward displacement of H8 (red arrow). This motion resulted in potential polar interactions including salt bridges between positively charged residues in H8 and a negatively charged side chains in TM7 (Asp298) below to the sodium binding pocket (shown in **A**). In the template structure (**C**, blue backbone) H8 localization and the position of residue 7.49 are similar to the commonly observed features in determined class A GPCR structures.

The straight TM5 conformation is maintained in 5 μs unbiased MD simulations of the hMC4R model. On the cytoplasmic side, sodium spontaneously binds to a site defined by Ser58^1.46^, Asp90^2.50^, Ser295^7.46^, and Asn294^7.45^ (**Figures 1**, **2**). Binding of sodium to the highly conserved Asp^2.50^ has also been observed in the NMR structure of the MC4R TM2 (Yun et al., 2015) and in the crystal structures of the protease-activated receptors 1 and 2 (Zhang et al., 2012; Cheng et al., 2017), as well as in the high-resolution structure of the A2A adenosine receptor (**Figure 2**) (Liu et al., 2012). Finally, a 5–6 Å outward movement of the distal end of TM7 relative to the template structure was observed, accompanied by a displacement of helix 8 (H8). H8 swivels inwards between TM1 and TM7 to be fixed by polar interactions, including a salt bridge between K314^8.55^ of H8 and Asp298^7.49^ of TM7 (**Figure 2**).

Of note, the crystallization construct used for structure determination of the S1PR1 template is characterized by three stabilizing mutations located directly at the interface of the TM7-H8 junction, at residues Lys250^6.29^, Ser251^6.30^, and Leu252^6.31^. To test whether these substitutions cause the observed differences between the S1PR1-template structure and the final hMC4R model, 2 μs MD simulations were performed on the S1P1R crystal structure with the original wild-type amino acids at these three positions. We find that the S1PR1 wild-type model indeed features high structural flexibility within this region, with similar RMSD to that observed in the hMC4R model (>5 Å RMSD, Supplementary Figure S2).

### Multi-Step Binding of the Antagonist MCL0129 to the hMC4R Model

In the 5 μs unbiased MD simulations described above, one MCL0129 molecule was placed in the extracellular solvent phase approximately 7 Å from the hMC4R surface. A funnel-like restraint centered on the receptor [as described in reference (Saleh et al., 2017b)] was set to focus sampling of the ligand close to the binding pocket. It is worth noting that this work reports the first use of a funnel-like restraint, which has so far been used exclusively for metadynamics sampling, in successful binding using unbiased MD simulation (Limongelli et al., 2013; Troussicot et al., 2015).

**FIGURE 3:**
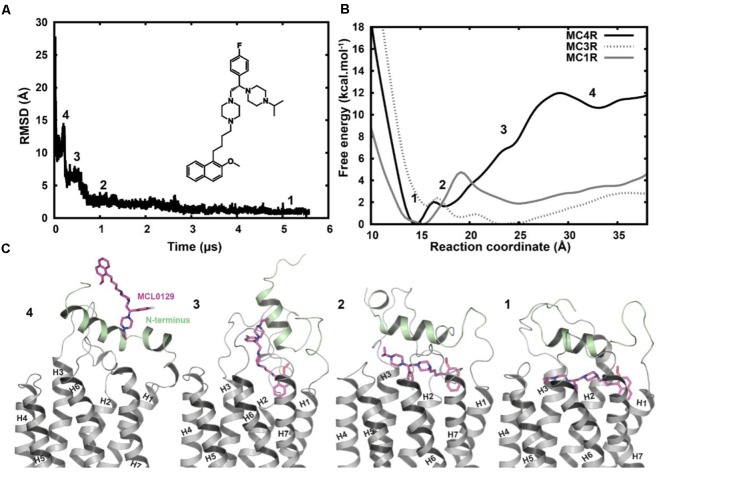
**(A)** Root-mean-square deviation of MCL0129 binding to hMC4R with a scheme for the structure of the non-peptidic antagonist MCL0129. **(B)** Free-energy profile for MCL0129 binding to hMC1R, hMC3R, and hMC4R with **(C)** representatives structures for the observed binding steps at MC4R (states 1–4).

Spontaneous binding of MCL0129 was observed to the ligand-binding pocket after ≈3 μs (**Figure 3**). The antagonist undergoes a four-step binding mechanism with three intermediate steps before settling at the hMC4R in its final position. Both the N-terminus (Nterm) and ECLs participate in recognition and spatial orientation of ligand binding. The highly flexible Nterm acts as a hook that traps the MCL0129 to guide it into the final binding mode, where the helical part of the Nterm caps the antagonist as a lid that prevents it leaving the pocket.

**FIGURE 4:**
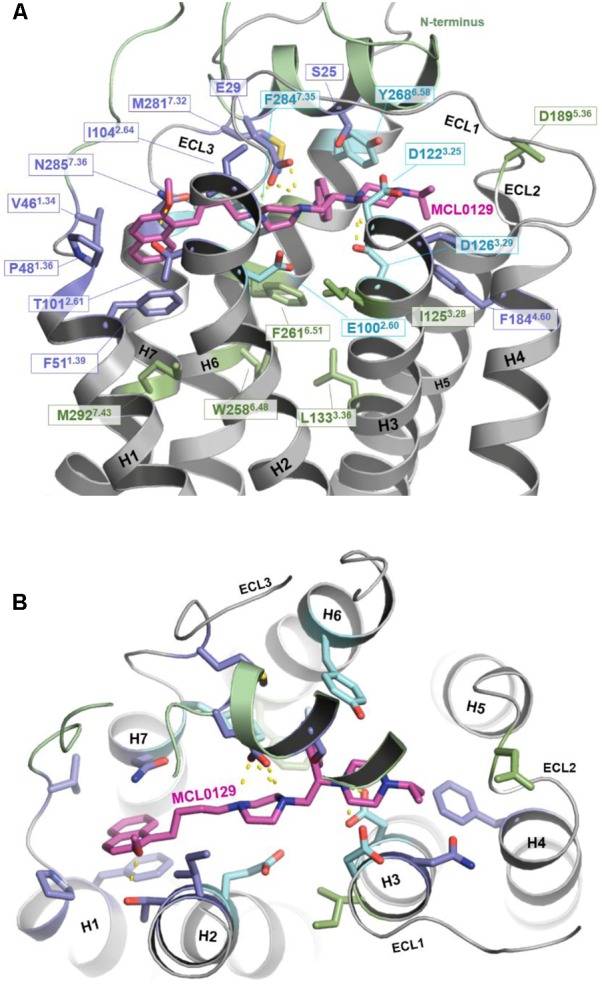
Binding mode of MCL0129. **(A)** Side view showing MCL0129 in magenta and potentially contacting residues as blue and cyan sticks. Residues identified by mutagenesis to interact specifically with agonists are indicated in green. Those side chains reported to interact with agonist and overlap with here suggested MCL0129 interactions are indicated cyan. **(B)** Top view for the binding mode of MCL0129-hMC4R.

In order to verify the final binding mode of MCL0129 obtained from 5 μs unbiased MD simulation, we used metadynamics simulation of 2 μs collective sampling. We followed the recently published funnel-metadynamics-based protocol (Saleh et al., 2017b) to characterize the global minimum for the hMC4R-MCL0129 complex. The metadynamics simulation was run independently from the unbiased MD simulation, using only the final pose of the MCL0129-hMC4R as starting geometry. We find that the final binding mode of MCL0129 obtained from unbiased MD simulation corresponds to the global minimum with ΔG_binding_ = -11.5 ± 0.3 kcal mol^-1^ (**Figure 3**) and K_i_ ≈ 6.5 nM [experimental K_i_ = 7.99 nM (Pontillo et al., 2005)]. This binding mode (state 1) is defined by contacts of MCL0129 to amino acids from the Nterm, ECL1-3 and TM1-7 except for TM5 (**Figures 4A,B** and Supplementary Figure S3). Specifically, hydrogen bonds are formed with Glu29^Nterm^, Thr101^2.61^, and Asp126^3.25^, in addition to aromatic interactions with Phe51^1.39^, Phe284^7.35^, and Tyr268^6.58^ (**Figure 4** and Supplementary Table S1).

### Antagonist Versus Agonist Interactions at hMC4R

The binding mode of MCL0129 matches experimentally identified interactions of peptidic ligands, including the endogenous inverse agonist AgRP with MC4R (Yang et al., 2000; Nickolls et al., 2003; Tao and Segaloff, 2003; Chen et al., 2007; Tao, 2010). The acidic residues Asp122^3.25^ and Asp126^3.29^, which have been predicted to be directly involved in AgRP binding (Yang et al., 2000; Nickolls et al., 2003; Tao and Segaloff, 2003; Chen et al., 2007), form an ionic interaction with the positively-charged piperazine moiety of MCL0129 in a sandwich-like mode (one above and one below).

Comparison of the MCL0129 antagonist binding site with the proposed agonist-binding site (reviewed in Tao, 2010; Ericson et al., 2017a) provides an explanation for the different pharmacological effects. The spatial overlap of binding at the extracellular sides of TM1, TM2, TM3, and TM7 (**Figure 4**) suggests that MCL0129 prevents access of the agonist to the binding pocket, and thus acts by direct competition. MCL0129, however, does not interact with the specific residues Ile125^3.28^, Leu133^3.36^, Trp258^6.48^, Phe261^6.51^, and Met292^7.43^ (**Figure 4**) from the putative agonist-binding pocket (Yang et al., 2000; Nickolls et al., 2003; Tao and Segaloff, 2003; Chen et al., 2007; Tao, 2010). We assume that these additional contacts located deeper in the TM bundle define agonist- vs. antagonist-ligand binding properties.

### MCL0129 Selectivity for MCR Subtypes

**FIGURE 5:**
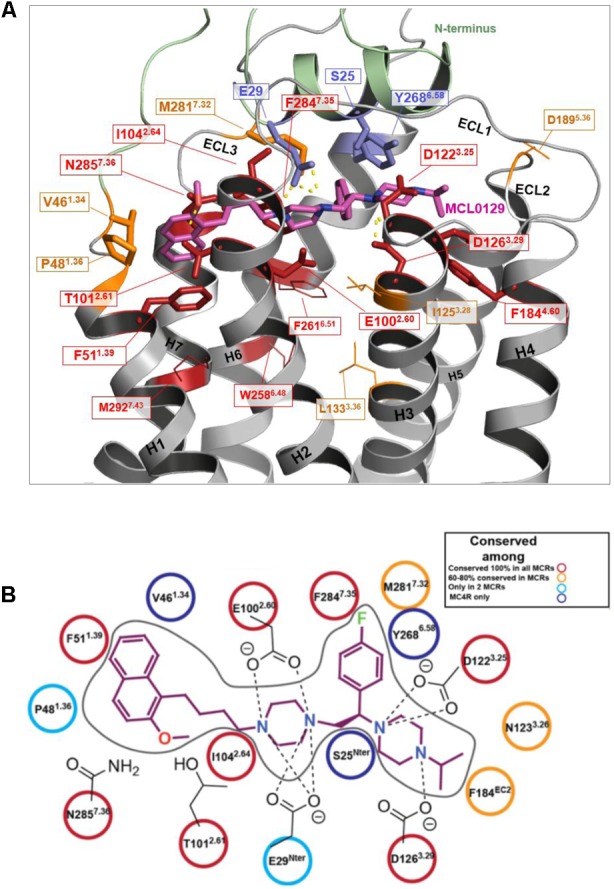
Conservation of ligand binding site residues among the hMCR group. **(A)** Red: highly conserved in sequence; Orange: low sequence conservation (same residue in one further hMCR subtype or maintained biophysical properties like hydrophobic side chains); Blue: hMC4R specific amino acids (no sequence conservation); Residues shown as sticks form potential contacts between hMC4R and MCL0129; Residues shown as lines are side chains suggested to be involved in action of agonists, but not for MCL0129 binding. **(B)** Interaction diagram for the MCL0129-hMC4R complex color-coded based on the conservation of these interacting residues among the melanocortin receptors.

Amino acid variations of the MCL0129 binding pocket between all five melanocortin receptors were analyzed to evaluate subtype selectivity of MCL0129 (Pontillo et al., 2005) (**Figure 5**). Briefly, three residues Ser25^Nterm^, Val46^1.34^, and Tyr268^6.58^ are found exclusively in hMC4R, while residues Glu29^Nterm^ and Pro48^1.36^ are shared between hMC4R and MC2R or MC3R, respectively (Supplementary Figure S1). To test whether these differences contribute to subtype selectivity, we analyzed MCL0129 binding to hMC1R and hMC3R by an additional set of MD simulations. For this purpose, hMC1R and hMC3R subtype models were built based on the refined hMC4R structure obtained after 5 μs MD simulations. Next, these subtype models were simulated with the final docking pose of MCL0129 obtained for hMC4R for 2 μs of unbiased MD simulation each. The high RMSD values of 7 and 13 Å observed for the MCL0129 ligand with hMC1R and hMC3R, respectively, reveal high positional variability, indicating lower affinity to the MCL0129 (Supplementary Figure S2B). The subsequent metadynamics simulations highlight that MCL0129 indeed binds very weakly (with binding free energies less than -5 kcal mol^-1^ for hMC1R and hMC3R, respectively), in agreement with the experimental observation that neither subtype binds MCL0129 up to 10 μM (Pontillo et al., 2005).

## Discussion

This study was intended to gain insight into the structure and specific ligand-binding properties of MC4R, a high-priority drug target for treating obesity (Zhou and Cai, 2017; Ju et al., 2018). In the absence of an experimental structure, we used microsecond-scale MD simulations to study binding of the non-peptide antagonist MCL0129 to a homology model of human MC4R (hMC4R). To elucidated subtype selectivity, we evaluated the binding properties of MCL0129 to structural models of the homologous hMC1R and hMC3R receptors.

The initial hMC4R model is based on the crystal structure of the homologous sphingosine 1-phosphate receptor 1 (Hanson et al., 2012), which is characterized by the absence of a proline-induced kink in TM5 and a disulfide bridge between TM3 and ECL2. The absence of Pro^5.50^ causes a regular α-helical conformation different from other GPCRs that have a kink and a bulge in TM5 (Pro^5.50^ is approximately 80% conserved) (Sansuk et al., 2011). The 5 μs unbiased MD simulations of the hMC4R suggest that this straight TM5 conformation is maintained. The regular α-helical conformation of TM5 particularly defines the residues that form the putative ligand-binding site suggested here (**Figure 3**). A different set and spatial localization of residues would become accessible for interaction with the ligand in a kinked and slightly rotated TM5.

Moreover, we identified a sodium-binding site defined by amino acid residues in TM 1, 2 and 7, which is known to impact ligand binding (allosteric effects) and protein stability (Liu et al., 2012). Key players in this motif are amino acids Ser58^1.46^, Asp90^2.50^, Ser295^7.46^, and Asn294^7.45^ (**Figure 2**). This observation is in general agreement with NMR data on MC4R and crystal structures of other GPCRs (Zhang et al., 2012; Yun et al., 2015; Cheng et al., 2017), with the difference that the putative hMC4R sodium-binding site is located between TM2 and TM7, close to TM1, while in the other GPCRs it is more located towards TM3.

Finally, we find that in our simulations, a characteristic outward movement of TM7 by a 5–6 Å in the distal end relative to the template or other GPCR crystal structures. This novel structural feature is accompanied by a displacement of H8 in-between TM1 and TM7 (**Figure 2**), which indicates high flexibility of TM7 and H8. In agreement, in the homologous template structure this part was thermo-stabilized by site-directed mutations (Supplementary Figure S2).

In accordance with previous computational and experimental work carried out for other receptors (Dror et al., 2011; Bock et al., 2012; Kruse et al., 2012; DeVree et al., 2016; Milanos et al., 2016; Saleh et al., 2016, 2017a,c), our study suggests a stepwise binding mechanism for the selective non-peptide antagonist MCL0129 to hMC4R. The Nterm plays a key role in ligand binding and recognition, trapping MCL0129 into its final binding mode.

This binding side of MCL0129 is defined by contacts with ECL1-3 and TM1-4 and TM6-7, for which we assume a straight helical conformation in the absence of Pro^5.50^. MCL0129 is stabilized by hydrogen bonds with Glu29^Nterm^, Thr101^2.61^ and Asp126^3.25^, in addition to aromatic interactions with Phe51^1.39^, Phe284^7.35^, and Tyr268^6.58^ (**Figures 3**, **4** and Supplementary Table S1).

Partial overlap of the antagonist binding site with the proposed agonist binding site suggests that MCL0129 directly blocks the agonist binding (**Figure 4A**). In addition, several of the contacts proposed to be essential for agonist binding are missing in the hMC4R/antagonist complex, thus explaining the antagonistic properties at hMC4R. We identified several hMC4R residues as potential selectivity determinants between hMC4R and other hMCRs (three are hMC4R-specific and two are highly variant), located in both the 7-TM bundle and the Nterm. Such differences between MCR subtypes may affect future efforts to design selective ligands for the MCR’s.

Finally, the ability of MD simulations combined with free-energy calculations to track and identify intermediate steps of ligand binding has increasingly contributed to our understanding of GPCR-ligand interactions and helped to identify potential binding sites and strategies for SBDD, which remains challenging for many GPCRs that are still not therapeutically accessible. Our MC4R-related insights obtained from computational simulations may pave the way for rational ligand design in the field of medicinal chemistry targeting the melanocortin systems.

## Author Contributions

NS: project idea, modeling and simulation studies, data analysis, wrote the manuscript, and final approval. GK: project idea, modeling studies, data analysis, wrote the manuscript, and final approval. NH and TC: data analysis, wrote the manuscript, and final approval. PH and PS project idea and coordination, data analysis, wrote the manuscript, and final approval.

## Conflict of Interest Statement

The authors declare that the research was conducted in the absence of any commercial or financial relationships that could be construed as a potential conflict of interest.

## References

[B1] Ballesteros J. A. Weinstein H. (1995). Integrated methods for the construction of three-dimensional models and computational probing of structure-function relationships in G-protein coupled receptors. *Methods Neurosci.* 25 366–428. 10.1016/S1043-9471(05)80049-7

[B2] Barducci A. Bussi G. Parrinello M. (2008). Well-tempered metadynamics: a smoothly converging and tunable free-energy method. *Phys. Rev. Lett.* 100:020603. 10.1103/PhysRevLett.100.020603 18232845

[B3] Bock A. Merten N. Schrage R. Dallanoce C. Batz J. Klockner J. (2012). The allosteric vestibule of a seven transmembrane helical receptor controls G-protein coupling. *Nat. Commun.* 3:1044. 10.1038/ncomms2028 22948826 PMC3658004

[B4] Chaki S. Hirota S. Funakoshi T. Suzuki Y. Suetake S. Okubo T. (2003). Anxiolytic-like and antidepressant-like activities of MCL0129 (1-[(S)-2-(4-fluorophenyl)-2-(4-isopropylpiperadin-1-yl)ethyl]-4-[4-(2-methoxynap hthalen-1-yl)butyl]piperazine), a novel and potent nonpeptide antagonist of the melanocortin-4 receptor. *J. Pharmacol. Exp. Ther.* 304 818–826. 10.1124/jpet.102.044826 12538838

[B5] Chaki S. Okubo T. (2007). Melanocortin-4 receptor antagonists for the treatment of depression and anxiety disorders. *Curr. Top. Med. Chem.* 7 1145–1151. 10.2174/156802607780906618 17584135

[B6] Chen K. Y. Muniyappa R. Abel B. S. Mullins K. P. Staker P. Brychta R. J. (2015). RM-493, a melanocortin-4 receptor (MC4R) agonist, increases resting energy expenditure in obese individuals. *J. Clin. Endocrinol. Metab.* 100 1639–1645. 10.1210/jc.2014-4024 25675384 PMC4399297

[B7] Chen M. Cai M. Aprahamian C. J. Georgeson K. E. Hruby V. Harmon C. M. (2007). Contribution of the conserved amino acids of the melanocortin-4 receptor in [corrected] [Nle4,D-Phe7]-alpha-melanocyte-stimulating [corrected] hormone binding and signaling. *J. Biol. Chem.* 282 21712–21719. 10.1074/jbc.M702285200 17545153 PMC2704061

[B8] Chen W. Kelly M. A. Opitz-Araya X. Thomas R. E. Low M. J. Cone R. D. (1997). Exocrine gland dysfunction in MC5-R-deficient mice: evidence for coordinated regulation of exocrine gland function by melanocortin peptides. *Cell* 91 789–798. 10.1016/S0092-8674(00)80467-5 9413988

[B9] Cheng R. K. Y. Fiez-Vandal C. Schlenker O. Edman K. Aggeler B. Brown D. G. (2017). Structural insight into allosteric modulation of protease-activated receptor 2. *Nature* 545 112–115. 10.1038/nature22309 28445455

[B10] Clark T. (2017). G-Protein coupled receptors: answers from simulations. *Beilstein J. Org. Chem.* 13 1071–1078. 10.3762/bjoc.13.106 28684986 PMC5480328

[B11] Cone R. D. (2005). Anatomy and regulation of the central melanocortin system. *Nat. Neurosci.* 8 571–578. 10.1038/nn1455 15856065

[B12] Cone R. D. (2006). Studies on the physiological functions of the melanocortin system. *Endocr. Rev.* 27 736–749. 10.1210/er.2006-0034 17077189

[B13] Costanzi S. Skorski M. Deplano A. Habermehl B. Mendoza M. Wang K. (2016). Homology modeling of a Class A GPCR in the inactive conformation: a quantitative analysis of the correlation between model/template sequence identity and model accuracy. *J. Mol. Graph. Model.* 70 140–152. 10.1016/j.jmgm.2016.10.004 27723562 PMC5138091

[B14] Darden T. York D. Pedersen L. (1993). Particle mesh Ewald – an N.Log(N) method for Ewald sums in large systems. *J. Chem. Phys.* 98 10089–10092. 10.1063/1.464397

[B15] DeVree B. T. Mahoney J. P. Velez-Ruiz G. A. Rasmussen S. G. Kuszak A. J. Edwald E. (2016). Allosteric coupling from G protein to the agonist-binding pocket in GPCRs. *Nature* 535 182–186. 10.1038/nature18324 27362234 PMC5702553

[B16] Dores R. M. Londraville R. L. Prokop J. Davis P. Dewey N. Lesinski N. (2014). Molecular evolution of GPCRs: melanocortin/melanocortin receptors. *J. Mol. Endocrinol.* 52 T29–T42. 10.1530/JME-14-0050 24868105

[B17] Dorr R. T. Lines R. Levine N. Brooks C. Xiang L. Hruby V. J. (1996). Evaluation of melanotan-II, a superpotent cyclic melanotropic peptide in a pilot phase-I clinical study. *Life Sci.* 58 1777–1784. 10.1016/0024-3205(96)00160-9 8637402

[B18] Dror R. O. Dirks R. M. Grossman J. P. Xu H. Shaw D. E. (2012). Biomolecular simulation: a computational microscope for molecular biology. *Annu. Rev. Biophys.* 41 429–452. 10.1146/annurev-biophys-042910-155245 22577825

[B19] Dror R. O. Green H. F. Valant C. Borhani D. W. Valcourt J. R. Pan A. C. (2013). Structural basis for modulation of a G-protein-coupled receptor by allosteric drugs. *Nature* 503 295–299. 10.1038/nature12595 24121438

[B20] Dror R. O. Pan A. C. Arlow D. H. Borhani D. W. Maragakis P. Shan Y. (2011). Pathway and mechanism of drug binding to G-protein-coupled receptors. *Proc. Natl. Acad. Sci. U.S.A.* 108 13118–13123. 10.1073/pnas.1104614108 21778406 PMC3156183

[B21] Enriori P. J. Chen W. Garcia-Rudaz M. C. Grayson B. E. Evans A. E. Comstock S. M. (2016). alpha-Melanocyte stimulating hormone promotes muscle glucose uptake via melanocortin 5 receptors. *Mol. Metab.* 5 807–822. 10.1016/j.molmet.2016.07.009 27688995 PMC5034615

[B22] Ercil N. E. Galici R. Kesterson R. A. (2005). HS014, a selective melanocortin-4 (MC4) receptor antagonist, modulates the behavioral effects of morphine in mice. *Psychopharmacology (Berl)* 180 279–285. 10.1007/s00213-005-2166-x 15719225

[B23] Ericson M. D. Lensing C. J. Fleming K. A. Schlasner K. N. Doering S. R. Haskell-Luevano C. (2017a). Bench-top to clinical therapies: a review of melanocortin ligands from 1954 to 2016. *Biochim. Biophys. Acta* 1863 2414–2435. 10.1016/j.bbadis.2017.03.020 28363699 PMC5600687

[B24] Ericson M. D. Lensing C. J. Fleming K. A. Schlasner K. N. Doering S. R. Haskell-Luevano C. (2017b). Bench-top to clinical therapies: a review of melanocortin ligands from 1954 to 2016. *Biochim. Biophys. Acta* 1863(10 Pt A), 2414–2435. 10.1016/j.bbadis.2017.03.020 28363699 PMC5600687

[B25] Farooqi I. S. Yeo G. S. O’rahilly S. (2003). Binge eating as a phenotype of melanocortin 4 receptor gene mutations. *N. Engl. J. Med.* 349 606–609 author reply 606–609 10.1056/NEJM200308073490615 12908459

[B26] Fridmanis D. Roga A. Klovins J. (2017). ACTH receptor (MC2R) specificity: what do we know about underlying molecular mechanisms? *Front. Endocrinol. (Lausanne)* 8:13. 10.3389/fendo.2017.00013 28220105 PMC5292628

[B27] Gantz I. Miwa H. Konda Y. Shimoto Y. Tashiro T. Watson S. J. (1993). Molecular cloning, expression, and gene localization of a fourth melanocortin receptor. *J. Biol. Chem.* 268 15174–15179. 8392067

[B28] Girardet C. Begriche K. Ptitsyn A. Koza R. A. Butler A. A. (2014). Unravelling the mysterious roles of melanocortin-3 receptors in metabolic homeostasis and obesity using mouse genetics. *Int. J. Obes. Suppl.* 4 S37–S44. 10.1038/ijosup.2014.10 27152165 PMC4850579

[B29] Goncalves J. P. L. Palmer D. Meldal M. (2018). MC4R agonists: structural overview on antiobesity therapeutics. *Trends Pharmacol. Sci.* 39 402–423. 10.1016/j.tips.2018.01.004 29478721

[B30] Granier S. Kobilka B. (2012). A new era of GPCR structural and chemical biology. *Nat. Chem. Biol.* 8 670–673. 10.1038/nchembio.1025 22810761 PMC4031315

[B31] Grossfield A. (2011). Recent progress in the study of G protein-coupled receptors with molecular dynamics computer simulations. *Biochim. Biophys. Acta* 1808 1868–1878. 10.1016/j.bbamem.2011.03.010 21443858

[B32] Hanson M. A. Roth C. B. Jo E. Griffith M. T. Scott F. L. Reinhart G. (2012). Crystal structure of a lipid G protein-coupled receptor. *Science* 335 851–855. 10.1126/science.1215904 22344443 PMC3338336

[B33] Herraiz C. Garcia-Borron J. C. Jimenez-Cervantes C. Olivares C. (2017). MC1R signaling. Intracellular partners and pathophysiological implications. *Biochim. Biophys. Acta* 1863 2448–2461. 10.1016/j.bbadis.2017.02.027 28259754

[B34] Huang J. MacKerell A. D. Jr (2013). CHARMM36 all-atom additive protein force field: validation based on comparison to NMR data. *J. Comput. Chem.* 34 2135–2145. 10.1002/jcc.23354 23832629 PMC3800559

[B35] Jakalian A. Jack D. B. Bayly C. I. (2002). Fast, efficient generation of high-quality atomic charges. AM1-BCC model: II. Parameterization and validation. *J. Comput. Chem.* 23 1623–1641. 10.1002/jcc.10128 12395429

[B36] Jo S. Kim T. Iyer V. G. Im W. (2008). CHARMM-GUI: a web-based graphical user interface for CHARMM. *J. Comput. Chem.* 29 1859–1865. 10.1002/jcc.20945 18351591

[B37] Ju S. H. Cho G. B. Sohn J. W. (2018). Understanding melanocortin-4 receptor control of neuronal circuits: toward novel therapeutics for obesity syndrome. *Pharmacol. Res.* 129 10–19. 10.1016/j.phrs.2018.01.004 29329999

[B38] Kievit P. Halem H. Marks D. L. Dong J. Z. Glavas M. M. Sinnayah P. (2013). Chronic treatment with a melanocortin-4 receptor agonist causes weight loss, reduces insulin resistance, and improves cardiovascular function in diet-induced obese rhesus macaques. *Diabetes* 62 490–497. 10.2337/db12-0598 23048186 PMC3554387

[B39] Kruse A. C. Hu J. Pan A. C. Arlow D. H. Rosenbaum D. M. Rosemond E. (2012). Structure and dynamics of the M3 muscarinic acetylcholine receptor. *Nature* 482 552–556. 10.1038/nature10867 22358844 PMC3529910

[B40] Kuhnen P. Clement K. Wiegand S. Blankenstein O. Gottesdiener K. Martini L. L. (2016). Proopiomelanocortin deficiency treated with a melanocortin-4 receptor agonist. *N. Engl. J. Med.* 375 240–246. 10.1056/NEJMoa1512693 27468060

[B41] Laio A. Gervasio F. L. (2008). Metadynamics: a method to simulate rare events and reconstruct the free energy in biophysics, chemistry and material science. *Rep. Prog. Phys.* 71:126601 10.1088/0034-4885/71/12/126601

[B42] Laio A. Parrinello M. (2002). Escaping free-energy minima. *Proc. Natl. Acad. Sci. U.S.A.* 99 12562–12566. 10.1073/pnas.202427399 12271136 PMC130499

[B43] Lansdell M. I. Hepworth D. Calabrese A. Brown A. D. Blagg J. Burring D. J. (2010). Discovery of a selective small-molecule melanocortin-4 receptor agonist with efficacy in a pilot study of sexual dysfunction in humans. *J. Med. Chem.* 53 3183–3197. 10.1021/jm9017866 20329799

[B44] Laskowski R. A. Moss D. S. Thornton J. M. (1993). Main-chain bond lengths and bond angles in protein structures. *J. Mol. Biol.* 231 1049–1067. 10.1006/jmbi.1993.1351 8515464

[B45] Lee J. Cheng X. Swails J. M. Yeom M. S. Eastman P. K. Lemkul J. A. (2016). CHARMM-GUI input generator for NAMD, GROMACS, AMBER, OpenMM, and CHARMM/OpenMM simulations using the CHARMM36 additive force field. *J. Chem. Theory Comput.* 12 405–413. 10.1021/acs.jctc.5b00935 26631602 PMC4712441

[B46] Liang L. Angleson J. K. Dores R. M. (2013). Using the human melanocortin-2 receptor as a model for analyzing hormone/receptor interactions between a mammalian MC2 receptor and ACTH(1-24). *Gen. Comp. Endocrinol.* 181 203–210. 10.1016/j.ygcen.2012.11.011 23201148

[B47] Limongelli V. Bonomi M. Parrinello M. (2013). Funnel metadynamics as accurate binding free-energy method. *Proc. Natl. Acad. Sci. U.S.A.* 110 6358–6363. 10.1073/pnas.1303186110 23553839 PMC3631651

[B48] Liu W. Chun E. Thompson A. A. Chubukov P. Xu F. Katritch V. (2012). Structural basis for allosteric regulation of GPCRs by sodium ions. *Science* 337 232–236. 10.1126/science.1219218 22798613 PMC3399762

[B49] Lomize M. A. Lomize A. L. Pogozheva I. D. Mosberg H. I. (2006). OPM: orientations of proteins in membranes database. *Bioinformatics* 22 623–625. 10.1093/bioinformatics/btk023 16397007

[B50] Milanos L. Saleh N. Kling R. C. Kaindl J. Tschammer N. Clark T. (2016). Identification of two distinct sites for antagonist and biased agonist binding to the human chemokine receptor CXCR3. *Angew. Chem. Int. Ed. Engl.* 55 15277–15281. 10.1002/anie.201607831 27862735

[B51] Mountjoy K. G. (2015). Pro-Opiomelanocortin (POMC) neurones, POMC-derived peptides, melanocortin receptors and obesity: how understanding of this system has changed over the last decade. *J. Neuroendocrinol.* 27 406–418. 10.1111/jne.12285 25872650

[B52] Nickolls S. A. Cismowski M. I. Wang X. Wolff M. Conlon P. J. Maki R. A. (2003). Molecular determinants of melanocortin 4 receptor ligand binding and MC4/MC3 receptor selectivity. *J. Pharmacol. Exp. Ther.* 304 1217–1227. 10.1124/jpet.102.044974 12604699

[B53] Pontillo J. Tran J. A. Markison S. Joppa M. Fleck B. A. Marinkovic D. (2005). A potent and selective nonpeptide antagonist of the melanocortin-4 receptor induces food intake in satiated mice. *Bioorg. Med. Chem. Lett.* 15 2541–2546. 10.1016/j.bmcl.2005.03.053 15863313

[B54] Pronk S. Pall S. Schulz R. Larsson P. Bjelkmar P. Apostolov R. (2013). GROMACS 4.5: a high-throughput and highly parallel open source molecular simulation toolkit. *Bioinformatics* 29 845–854. 10.1093/bioinformatics/btt055 23407358 PMC3605599

[B55] Raiteri P. Laio A. Gervasio F. L. Micheletti C. Parrinello M. (2006). Efficient reconstruction of complex free energy landscapes by multiple walkers metadynamics. *J. Phys. Chem. B* 110 3533–3539. 10.1021/jp054359r 16494409

[B56] Rodrigues A. R. Almeida H. Gouveia A. M. (2015). Intracellular signaling mechanisms of the melanocortin receptors: current state of the art. *Cell. Mol. Life Sci.* 72 1331–1345. 10.1007/s00018-014-1800-3 25504085 PMC11113477

[B57] Rose A. S. Elgeti M. Zachariae U. Grubmuller H. Hofmann K. P. Scheerer P. (2014). Position of transmembrane helix 6 determines receptor G protein coupling specificity. *J. Am. Chem. Soc.* 136 11244–11247. 10.1021/ja5055109 25046433

[B58] Sabban E. L. Serova L. I. (2018). Potential of intranasal neuropeptide Y (NPY) and/or melanocortin 4 receptor (MC4R) antagonists for preventing or treating PTSD. *Mil. Med.* 183 408–412. 10.1093/milmed/usx228 29635611

[B59] Saleh N. Ibrahim P. Clark T. (2017a). Differences between G-protein-stabilized agonist-GPCR complexes and their nanobody-stabilized equivalents. *Angew. Chem. Int. Ed. Engl.* 56 9008–9012. 10.1002/anie.201702468 28481446

[B60] Saleh N. Ibrahim P. Saladino G. Gervasio F. L. Clark T. (2017b). An efficient metadynamics-based protocol to model the binding affinity and the transition state ensemble of G-protein-coupled receptor ligands. *J. Chem. Inf. Model.* 57 1210–1217. 10.1021/acs.jcim.6b00772 28453271

[B61] Saleh N. Saladino G. Gervasio F. L. Clark T. (2017c). Investigating allosteric effects on the functional dynamics of β2-adrenergic ternary complexes with enhanced-sampling simulations. *Chem. Sci.* 8 4019–4026. 10.1039/C6SC04647A 30155211 PMC6094175

[B62] Saleh N. Saladino G. Gervasio F. L. Haensele E. Banting L. Whitley D. C. (2016). A three-site mechanism for agonist/antagonist selective binding to vasopressin receptors. *Angew. Chem. Int. Ed. Engl.* 55 8008–8012. 10.1002/anie.201602729 27184628

[B63] Sansuk K. Deupi X. Torrecillas I. R. Jongejan A. Nijmeijer S. Bakker R. A. (2011). A structural insight into the reorientation of transmembrane domains 3 and 5 during family A G protein-coupled receptor activation. *Mol. Pharmacol.* 79 262–269. 10.1124/mol.110.066068 21081645

[B64] Tao Y. X. (2010). The melanocortin-4 receptor: physiology, pharmacology, and pathophysiology. *Endocr. Rev.* 31 506–543. 10.1210/er.2009-0037 20190196 PMC3365848

[B65] Tao Y.-X. Segaloff D. L. (2003). Functional characterization of melanocortin-4 receptor mutations associated with childhood obesity. *Endocrinology* 144 4544–4551. 10.1210/en.2003-0524 12959994

[B66] Tarnow P. Schoneberg T. Krude H. Gruters A. Biebermann H. (2003). Mutationally induced disulfide bond formation within the third extracellular loop causes melanocortin 4 receptor inactivation in patients with obesity. *J. Biol. Chem.* 278 48666–48673. 10.1074/jbc.M309941200 14504270

[B67] Tribello G. A. Bonomi M. Branduardi D. Camilloni C. Bussi G. (2014). Plumed 2: new feathers for an old bird. *Comput. Phys. Commun.* 185 604–613. 10.1016/j.cpc.2013.09.018

[B68] Troupiotis-Tsailaki A. Zachmann J. Gonzalez-Gil I. Gonzalez A. Ortega-Gutierrez S. Lopez-Rodriguez M. L. (2017). Ligand chain length drives activation of lipid G protein-coupled receptors. *Sci. Rep.* 7:2020. 10.1038/s41598-017-02104-5 28515494 PMC5435731

[B69] Troussicot L. Guilliere F. Limongelli V. Walker O. Lancelin J. M. (2015). Funnel-metadynamics and solution NMR to estimate protein-ligand affinities. *J. Am. Chem. Soc.* 137 1273–1281. 10.1021/ja511336z 25551252

[B70] Vanni S. Rothlisberger U. (2012). A closer look into G protein coupled receptor activation: X-ray crystallography and long-scale molecular dynamics simulations. *Curr. Med. Chem.* 19 1135–1145. 10.2174/092986712799320493 22300050

[B71] Vanommeslaeghe K. Hatcher E. Acharya C. Kundu S. Zhong S. Shim J. (2010). CHARMM general force field: a force field for drug-like molecules compatible with the CHARMM all-atom additive biological force fields. *J. Comput. Chem.* 31 671–690. 10.1002/jcc.21367 19575467 PMC2888302

[B72] Worth C. L. Kreuchwig F. Tiemann J. K. S. Kreuchwig A. Ritschel M. Kleinau G. (2017). GPCR-SSFE 2.0-a fragment-based molecular modeling web tool for Class A G-protein coupled receptors. *Nucleic Acids Res.* 45 W408–W415. 10.1093/nar/gkx399 28582569 PMC5570183

[B73] Wu E. L. Cheng X. Jo S. Rui H. Song K. C. Davila-Contreras E. M. (2014). CHARMM-GUI Membrane Builder toward realistic biological membrane simulations. *J. Comput. Chem.* 35 1997–2004. 10.1002/jcc.23702 25130509 PMC4165794

[B74] Yang L. K. Tao Y. X. (2017). Biased signaling at neural melanocortin receptors in regulation of energy homeostasis. *Biochim. Biophys. Acta* 863(10 Pt A), 2486–2495. 10.1016/j.bbadis.2017.04.010 28433713 PMC5600658

[B75] Yang Y. Harmon C. M. (2017). Molecular signatures of human melanocortin receptors for ligand binding and signaling. *Biochim. Biophys. Acta* 1863(10 Pt A), 2436–2447. 10.1016/j.bbadis.2017.04.025 28478228

[B76] Yang Y. K. Fong T. M. Dickinson C. J. Mao C. Li J. Y. Tota M. R. (2000). Molecular determinants of ligand binding to the human melanocortin-4 receptor. *Biochemistry* 39 14900–14911. 10.1021/bi001684q 11101306

[B77] Yun J. H. Kim M. Kim K. Lee D. Jung Y. Oh D. (2015). Solution structure of the transmembrane 2 domain of the human melanocortin-4 receptor in sodium dodecyl sulfate (SDS) micelles and the functional implication of the D90N mutant. *Biochim. Biophys. Acta* 1848 1294–1302. 10.1016/j.bbamem.2015.02.029 25753114

[B78] Zhang C. Srinivasan Y. Arlow D. H. Fung J. J. Palmer D. Zheng Y. (2012). High-resolution crystal structure of human protease-activated receptor 1. *Nature* 492 387–392. 10.1038/nature11701 23222541 PMC3531875

[B79] Zhou Y. Cai M. (2017). Novel approaches to the design of bioavailable melanotropins. *Expert Opin. Drug Discov.* 12 1023–1030. 10.1080/17460441.2017.1351940 28699792 PMC5953761

