# Supplementary material for: Binding, Thermodynamics, and Selectivity of a Non-peptide Antagonist to the Melanocortin-4 Receptor
Source: Front Pharmacol. 2018 Jun 1;9:560. doi: 10.3389/fphar.2018.00560 (PMC5992272; doi:10.3389/fphar.2018.00560)
Supplement: Supplementary file 1 [file Data_Sheet_1.doc]

***Supplemental Material***

Binding, thermodynamics and selectivity of a non-peptide antagonist to the Melanocortin-4 receptor

Noureldin Saleh ^1, a, 2, §^, *, Gunnar Kleinau ^1, b, §^, Nicolas Heyder ^1, b^, Timothy Clark ^3^,

Peter W. Hildebrand ^1, a, 4^, *, and Patrick Scheerer ^1, b^, *

^1^ Charité – Universitätsmedizin Berlin, corporate member of Freie Universität Berlin, Humboldt-Universität zu Berlin, and Berlin Institute of Health, Institute of Medical Physics and Biophysics, D-10117 Berlin, Germany, ^a^ Computational modelling and dynamics of molecular complexes, ^b^ Group Protein X-ray Crystallography and Signal Transduction

^2^ *present address:* Section for Biomolecular Sciences, Biology department, Biocenter, University of Copenhagen, DK-2200 Copenhagen N, Denmark

^3^ Computer-Chemie-Centrum, Department of Chemistry and Pharmacy, Friedrich-Alexander-Universität Erlangen-Nürnberg, 91052 Erlangen, Germany

^4^ Universität Leipzig, Institute of Medical Physics and Biophysics, 04107 Leipzig, Germany

^§^ These authors contributed equally to this work.

* Correspondence and requests for materials should be addressed to:

Dr. Patrick Scheerer (E-mail: [patrick.scheerer@charite.de](mailto:patrick.scheerer@charite.de)), Prof. Dr. Peter W. Hildebrand (E-mail: peter.hildebrand@medizin.uni-leipzig.de) and Dr. Noureldin Saleh (E-Mail: noureldin.saleh@bio.ku.dk)


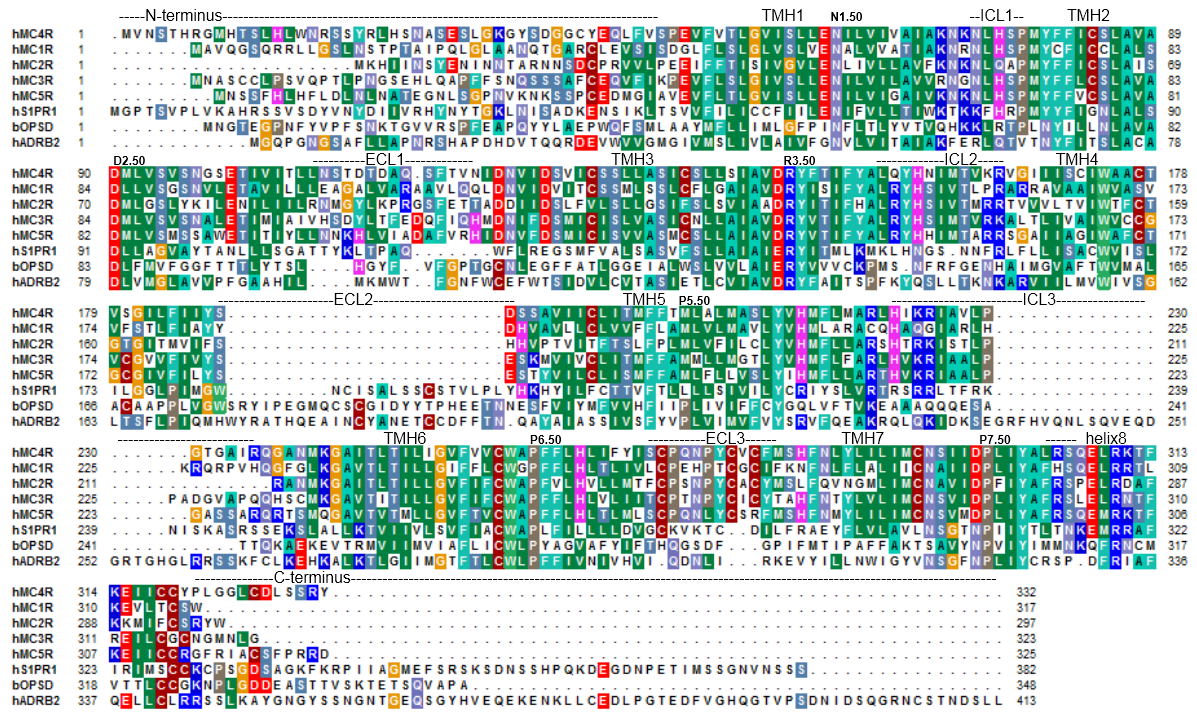
**Figure S1**

***Figure S1: Sequence comparison between hMCRs, the structural template S1PR1 and prototypical class A GPCRs.*** *This amino acid sequence alignment compares the hMC4R with further members of the hMCR group, the structural template (S1PR1 [1]) for MC4R modeling and the prototypical class A GPCRs rhodopsin (bovine, bOPSD) and human β-2 adrenergic receptor (hADRB2). Highlighted by color coding are similar biophysical side chain properties like hydrophobicity (green) or positively charged (blue) and negatively charged residues (red). In addition, the highly conserved amino acids according to the Ballesteros and Weinstein numbering scheme [2] are marked as well as putative structural dimensions of loop and helix regions predicted for hMCRs based on already determined GPCR structures [1; 3]. The sequence alignment was initially performed by using the software CLUSTALW and refined by manual corrections in the loop regions. Similarity and identity analyses was calculated by using the MATCH matrix and the BioEdit software was used for presentation.*

**Figure S2**

***/*a) b) c)**


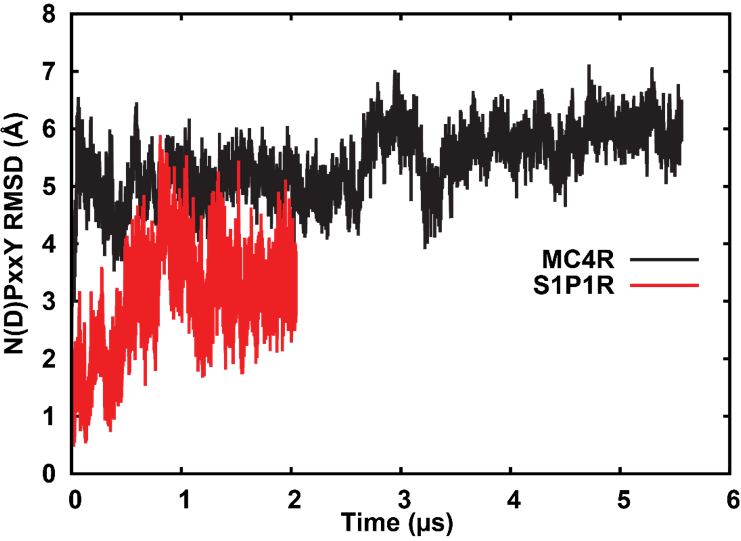

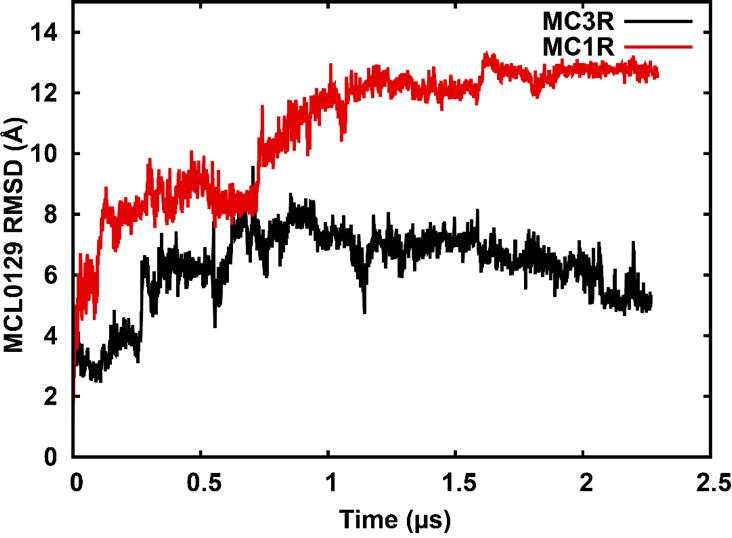

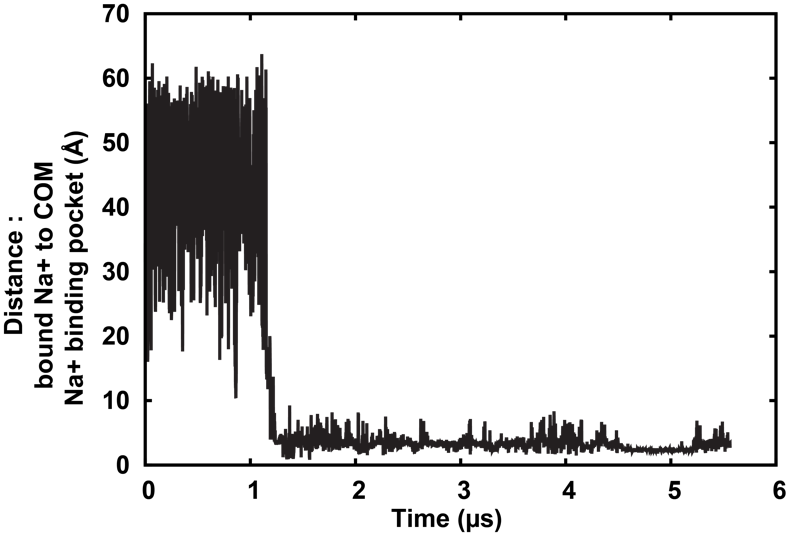


***Figure S2: (a) Comparison between the RMSD of the N(D)PxxY motif of S1P1R wild-type and MC4R.*** *Both receptors deviate at the region of this motif from the conformation of the S1P1R crystallographic construct;* ***(b) RMSD of MCL0129 bound to MC1R and MC3R.*** *Both receptors should low affinity to MCL0129’s pose observed with MC4R, marked by RMSD fluctuations of 13 and 7 Å respectively;* ***(c) Spontaneous binding of sodium ion.*** *Distance between the bound sodium ion and the center of mass of the polar side chains of residues S1.46 (S58, TM1), D2.50 (D90, TM2), S7.46 (S295, TM7) and N7.45 (N294, TM7).*

**Figure S3**


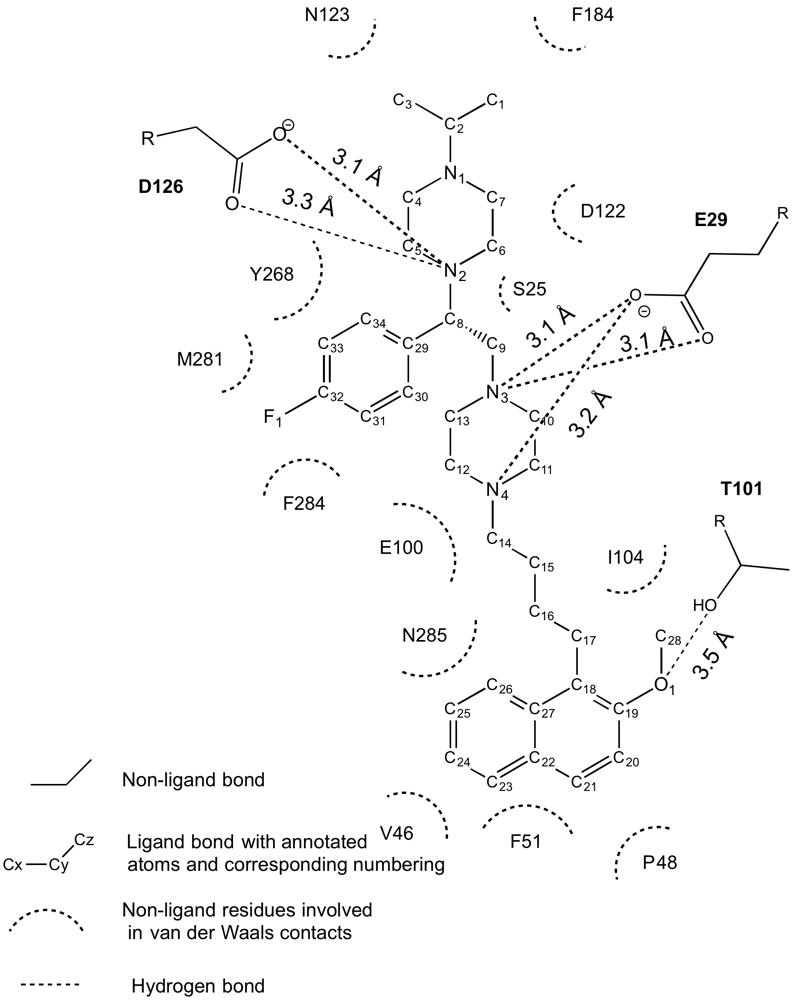
**a) b)**

| **MC4R amino acid** | **van der Waals contacts** |
| --- | --- |
| S25 | C8 |
| E29 | C8, C10-C13 |
|  | C29, C34 |
|  | N3-N4 |
| V46 | C21-C24 |
| P48 | C21 |
| F51 | C22-C27 |
| E100 | C12,13,15,17 |
| T101 | O1 |
|  | C18-C20 |
| I104 | C28, O1 |
| D122 | C6-C7 |
| N123 | C1 |
| D126 | C4-C9, C30 |
|  | N2 |
| F184 | C1 |
| Y268 | C4, C5, C33 |
| M281 | C33, F1 |
| F284 | C32, F1 |
| N285 | C16, C18  C24-27 |

***Figure S3: Interaction interface of hMC4R and antagonist MCL0129.*** ***(a)*** *The table summarizes potential van der Waals contact contacts between hMC4R and antagonist MCL0129 in the final binding mode.* ***(b)*** *Analysis of the potential hydrogen bonds and hydrophobic interactions between ligand receptor resulting from the final binding mode, including hydrophilic interactions (Table S1), are plotted in a scheme. In* ***(a)*** *and* ***(b)****, potential hydrogen bonds are indicated as black dashes with distances labels and were analysed using HBPLUS [4] with a maximum donor acceptor distance of 3.35 Å as implemented in the program LigPlot+ 1.45 [5]. Residues with closest distances less than 3.9 Å are considered to be in van der Waals contact and are shown as black dotted quarter circle.*

**Table S1**

| **Donor - Ligand** | **Acceptor - hMC4R** |  |  |
| --- | --- | --- | --- |
| **Atom** | **Amino acid** | **Atom** | **Distance [Å]** |
| N3 | Glu29 | OE2 | 3.1 |
| N4 | Glu29 | OE1 | 3.1 |
| N3 | Glu29 | OE1 | 3.2 |
| O1 | Thr101 | OG1 | 3.5 |
| N2 | Asp126 | OD1 | 3.1 |
| N2 | Asp126 | OD2 | 3.3 |

***Table S1: Potential hydrophilic contacts (hydrogen bonds) between hMC4R and antagonist MCL0129.*** *The final binding mode of antagonist MCL0129 suggests several potential hydrogen bonds with hMC4R.*

**REFERENCES**

[1] M.A. Hanson, C.B. Roth, E. Jo, M.T. Griffith, F.L. Scott, G. Reinhart, H. Desale, B. Clemons, S.M. Cahalan, S.C. Schuerer, M.G. Sanna, G.W. Han, P. Kuhn, H. Rosen, and R.C. Stevens, Crystal structure of a lipid G protein-coupled receptor. Science 335 (2012) 851-5.

[2] J.A. Ballesteros, and H. Weinstein, Integrated Methods for the Construction of Three-Dimensional Models and Computational Probing of Structure-Function Relationships in G-Protein Coupled Receptors. Methods Neurosci 25 (1995) 366-428.

[3] S.G. Rasmussen, H.J. Choi, D.M. Rosenbaum, T.S. Kobilka, F.S. Thian, P.C. Edwards, M. Burghammer, V.R. Ratnala, R. Sanishvili, R.F. Fischetti, G.F. Schertler, W.I. Weis, and B.K. Kobilka, Crystal structure of the human beta2 adrenergic G-protein-coupled receptor. Nature 450 (2007) 383-7.

[4] I.K. McDonald, and J.M. Thornton, Satisfying hydrogen bonding potential in proteins. Journal of molecular biology 238 (1994) 777-93.

[5] R.A. Laskowski, and M.B. Swindells, LigPlot+: multiple ligand-protein interaction diagrams for drug discovery. Journal of chemical information and modeling 51 (2011) 2778-86.
